# Pharmacological Analysis of Intrinsic Neuronal Oscillations in *rd10* Retina

**DOI:** 10.1371/journal.pone.0099075

**Published:** 2014-06-11

**Authors:** Sonia Biswas, Christine Haselier, Anja Mataruga, Gabriele Thumann, Peter Walter, Frank Müller

**Affiliations:** 1 Institute of Complex Systems, Cellular Biophysics, ICS-4, Forschungszentrum Jülich GmbH, Jülich, Germany; 2 Department of Ophthalmology, RWTH Aachen University, Aachen, Germany; The University of Melbourne, Australia

## Abstract

In the widely used mouse model of retinal degeneration, *rd1,* the loss of photoreceptors leads to rhythmic electrical activity of around 10–16 Hz in the remaining retinal network. Recent studies suggest that this oscillation is formed within the electrically coupled network of AII amacrine cells and ON-bipolar cells. A second mouse model, *rd10*, displays a delayed onset and slower progression of degeneration, making this mouse strain a better model for human retinitis pigmentosa. In *rd10*, oscillations occur at a frequency of 3–7 Hz, raising the question whether oscillations have the same origin in the two mouse models. As *rd10* is increasingly being used as a model to develop experimental therapies, it is important to understand the mechanisms underlying the spontaneous rhythmic activity. To study the properties of oscillations in *rd10* retina we combined multi electrode recordings with pharmacological manipulation of the retinal network. Oscillations were abolished by blockers for ionotropic glutamate receptors and gap junctions. Frequency and amplitude of oscillations were modulated strongly by blockers of inhibitory receptors and to a lesser extent by blockers of HCN channels. In summary, although we found certain differences in the pharmacological modulation of rhythmic activity in *rd10* compared to *rd1*, the overall pattern looked similar. This suggests that the generation of rhythmic activity may underlie similar mechanisms in *rd1* and *rd10* retina.

## Introduction

Retinitis pigmentosa (RP) is a genetically heterogeneous disease that leads to photoreceptor death associated with constriction of the visual field and, ultimately, blindness. A common reason for RP in humans is a mutation in the gene encoding the β-subunit of the rod phosphodiesterase (PDE). Similar mutations also exist in mouse, providing suitable animal models to investigate the course of retinal degeneration as well as therapeutic approaches.

Mouse models of retinal degeneration are indispensable tools to explore the mechanisms of degeneration as well as experimental therapies for the currently not treatable human RP. The most commonly used mouse model for RP is the *rd1* mouse [1]–[3]. In retinae of *rd1* mice, pronounced degeneration of photoreceptors starts around postnatal day 8 (P8). By three weeks of age, rods are completely lost [4], [5]. Cones also degenerate, albeit at a slower pace. Photoreceptor death leads to a total loss of the outer part of the retina, while the inner retinal part encompassing bipolar cells, horizontal cells, amacrine cells, and ganglion cells persists. However, recently evidence has accumulated that the death of photoreceptors leads to secondary remodelling of neurons in the inner retina. Remodelling includes loss or sprouting of neuronal processes, cell migration and reactive gliosis [6]–[9]. The fast degeneration in *rd1* mice is a major drawback of the model. Rod degeneration starts while the retina is still in the process of differentiation. Pathological processes observed in *rd1* retinae could, therefore, result from degeneration, a disturbed differentiation, or a mixture of both.

An alternative to *rd1* is the *rd10* mouse. These animals display a mutation in the same gene; however, the onset of photoreceptor degeneration is delayed. In *rd10* mice, rods start to degenerate after P16. By this time, all retinal layers and cell types as well as synaptic connections have been established and the major phase of retinal differentiation is over. Maximal cell death occurs between P21 and P25. By P60, only cones have survived. Hence, *rd10* mice mimic the disease process in human RP more accurately than *rd1* mice [10], [11].

Currently, there is no treatment for RP. However, the persistent inner retina provides a target for therapies. One possibility is driving retinal activity via electrical stimulation by neural prostheses or optical stimulation using ectopically expressed light sensitive proteins. In these attempts to restore vision, success crucially depends on the functional integrity of the remaining retinal ganglion cells and their ability to reliably transmit visual signals to the brain. In both *rd1* and *rd10*, spontaneous rhythmic electrical activity was observed in both ganglion cell spiking and in local field potentials recorded using multi electrode arrays (MEAs). The origin of this rhythmic electrical activity is not entirely clear. In *rd1* retinae, there is evidence that cone bipolar cells and AII amacrine cells may be involved [12]. However, while in *rd1* rhythmic activity displays frequencies in the range of 10–16 Hz, in *rd10* frequencies are lower [13]–[17]. Moreover, as the degeneration process differs between *rd1* and *rd10*, rhythmic activity might have different origins in both retinal models. It is important to understand the mechanisms and the properties of the rhythmic electrical activity as it may degrade the signal to noise ratio and may reduce the clarity of information transmission from eye to the brain. In the present study, we investigated the properties of rhythmic activity in *rd10* retinae using a pharmacological approach.

## Results

### Immunohistochemistry

**Figure 1:**
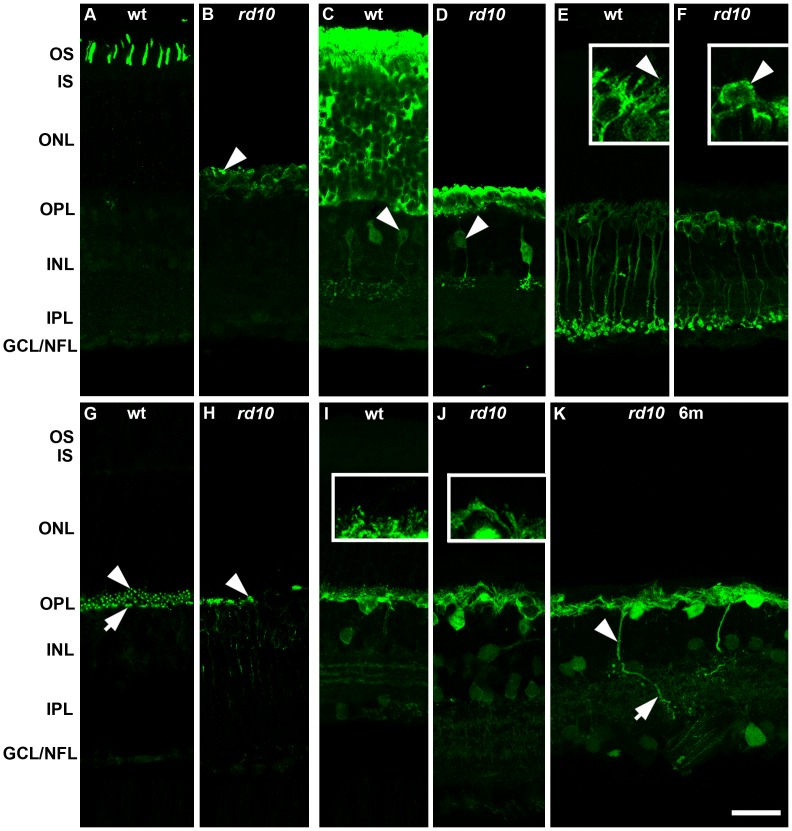
Comparison of confocal images of immunohistochemically stained vertical sections through the retinae of wild type mice (wt) and *rd10* mice. Stainings against red/green opsin (A, B), recoverin (C, D), PKCα (E, F), mGluR6 (G, H), and calbindin (I, J, K). With the exception of K, stainings of *rd10* retina are displayed at postnatal day 32 (P32). In contrast to wt (A) staining for red/green opsin in *rd10* was found in very few shortened outer segments (B, arrowhead) and somata of degenerating cone photoreceptors. The expression of recoverin in remaining photoreceptors and in type 2 cone bipolar cells of *rd10* (D) seemed to be unaffected compared to wt (C). In wt (E) rod bipolar cells were labeled with antibodies against PKCα (arrowhead indicates dendritic processes). In *rd10* (F) nearly all rod bipolar cell dendrites were lost at P32. Some PKCα-positive somata were displaced towards the outer retina (F, arrowhead). In wt (G) mGluR6 immunoreactivity was found as individual puncta (rod bipolar cell dendritic tips, arrowhead) and as condensed puncta at cone pedicles (cone ON-bipolar cell contacts, arrow). In *rd10*, only cone ON-bipolar cell contacts were detectable at P32 (H, arrowhead). In comparison to wt (I), horizontal cells in *rd10* retina extend much fewer fine dendrites (compare I and J, insets). Some horizontal cell bodies become displaced during degeneration (J, arrowhead). Ectopic processes of horizontal cells can be pronounced at later stages of degeneration (K, postnatal month 6). OS, outer segments; IS, inner segments; ONL, outer nuclear layer; OPL, outer plexiform layer; INL, inner nuclear layer; IPL, inner plexiform layer; GCL, ganglion cell layer; NFL, nerve fibre layer. Scale bars 25 µm for overviews and 10 µm for insets.

The degeneration of the outer retina, the changes in retinal thickness, and the retinal remodeling occurring in *rd10* mice have been analyzed by several groups using immunohistochemical stainings of vertical sections of different postnatal stages [10], [11]. Our immunohistochemical analysis in principle confirmed these recently published data. Although we carefully analyzed various postnatal stages (P6, P14, P20, P25, P32, P45, P60, and 6 months) using a variety of antibodies, only few stainings are shown in Fig. 1. In many instances we visualized proteins whose expression level differs strongly between different cell types or between different compartments of one cell, e.g. processes and soma. In order to visualize degeneration induced changes in the delicate and weakly labeled processes, sometimes saturation of the strongly stained structures (e.g. somata) had to be accepted. However, for each staining identical settings were chosen for wild type retina and *rd10* retina.

As published by others [10], [11] we observed an ongoing reduction in the number of photoreceptor cell layers in the ONL during maturation of the *rd10* retina (data not shown). While up to 12 rows are typical for an adult wild type retina and can still be found in *rd10* mice at postnatal stage 20, only up to 3 rows are detectable at P25. At P32, only one to two rows of somata in the ONL remained. In adult wild type retina, the antibody against red/green opsin stains numerous cone outer segments in the OS (Fig. 1A). In *rd10* retina (B) the morphology of these outer segments is clearly aberrant (arrowhead) and their number is considerably reduced.

In *rd10* retina at P32, the remaining photoreceptors are still highly immunoreactive for recoverin (D), similar as in wild type retina (C). In mouse retina, the antibody against recoverin also labels type 2 cone bipolar cells, albeit much weaker than photoreceptors [18]. As described earlier, the inner retinal cell types seem to be less affected in *rd10* mice than the outer retinal cells [10], [11]. At P32, we found intact type 2 cone bipolar cells that stratify in the correct sublamina of the IPL. At later stages however, somata were sometimes displaced and dendrites were missing (data not shown).

Compared to adult wild type mouse retina (E), rod bipolar cells of *rd10* mice stained with the antibody against PKCα (F) showed no obvious alteration in their somata, axons, and terminal systems. However, due to the ongoing degeneration of rod photoreceptors, rod bipolar cells revealed a clear reduction of their dendrites. At P32, most of the rods are already degenerated [11] and the loss of most of the rod bipolar cell dendrites in the OPL becomes obvious (compare inserts in E and F, arrowhead in E). Furthermore, some of the rod bipolar somata were located to an aberrant position between ONL and OPL (F, arrowhead).

In wild type, the antibody against mGluR6 stained two morphologically distinct structures. By double labeling with antibodies against PKCα (data not shown but see references) the cloud of fine puncta spreading from the OPL into the first row of somata in the ONL can be identified as dendritic tips of rod bipolar cells (punctate staining in G, arrowhead) [19], [20]. By double labeling with peanut agglutinin (data not shown) the mGluR6 positive dendritic tips of cone bipolar cells invaginating the cone pedicles can be identified [20]. As the dendritic tips are densely packed, at this magnification puncta merge into a flat continuously stained structure (arrow, G). In *rd10* P32 retinae, most of the punctate staining originating from rod bipolar dendritic tips had disappeared while mGluR6 at cone pedicles could still be observed. Moreover, mGluR6 staining had spread over the somata and axonal compartments of ON-bipolar cells (H).

Antibodies against calbindin stain the only horizontal cell type present in the mouse retina [21]. The somata of horizontal cells are located in the outermost row of the INL close to the OPL. While Gargini and co-workers [11] reported only slight changes in horizontal cells in *rd10*, more pronounced changes were described by Phillips and co-workers [10]. We, therefore, looked at changes in horizontal cells in more detail. At P32 we sometimes observed abnormally formed and misplaced horizontal cells in *rd10* retinae. In the example shown in Fig. 1J, the somata of some horizontal cells were shifted towards the middle of the INL (arrowhead). Compared to wild type (I, inset) the CabP-positive processes in the OPL were clearly reduced at P32 (J, inset) as most of the photoreceptors had disappeared at this stage. At later stages, sprouting of ectopic processes was regularly found in horizontal cells (Fig. 1K), confirming the report of Phillips et al. [10]. The much weaker stained CabP-positive amacrine cells in the INL and displaced amacrine cells in the GCL were found in wild type as well as in *rd10* retina. This was also true for the three fine layers in the IPL which are formed by the stratification of ON- and OFF-cholinergic and nitric oxide synthase-positive amacrine cells [22]. Thus, the general architecture of the inner retina, especially the IPL, barely seems to be changed at P32, but may be affected at later stages (Fig. 1K).

### Retinae of rd10 Mice Display Spontaneous Rhythmic Activity

**Figure 2:**
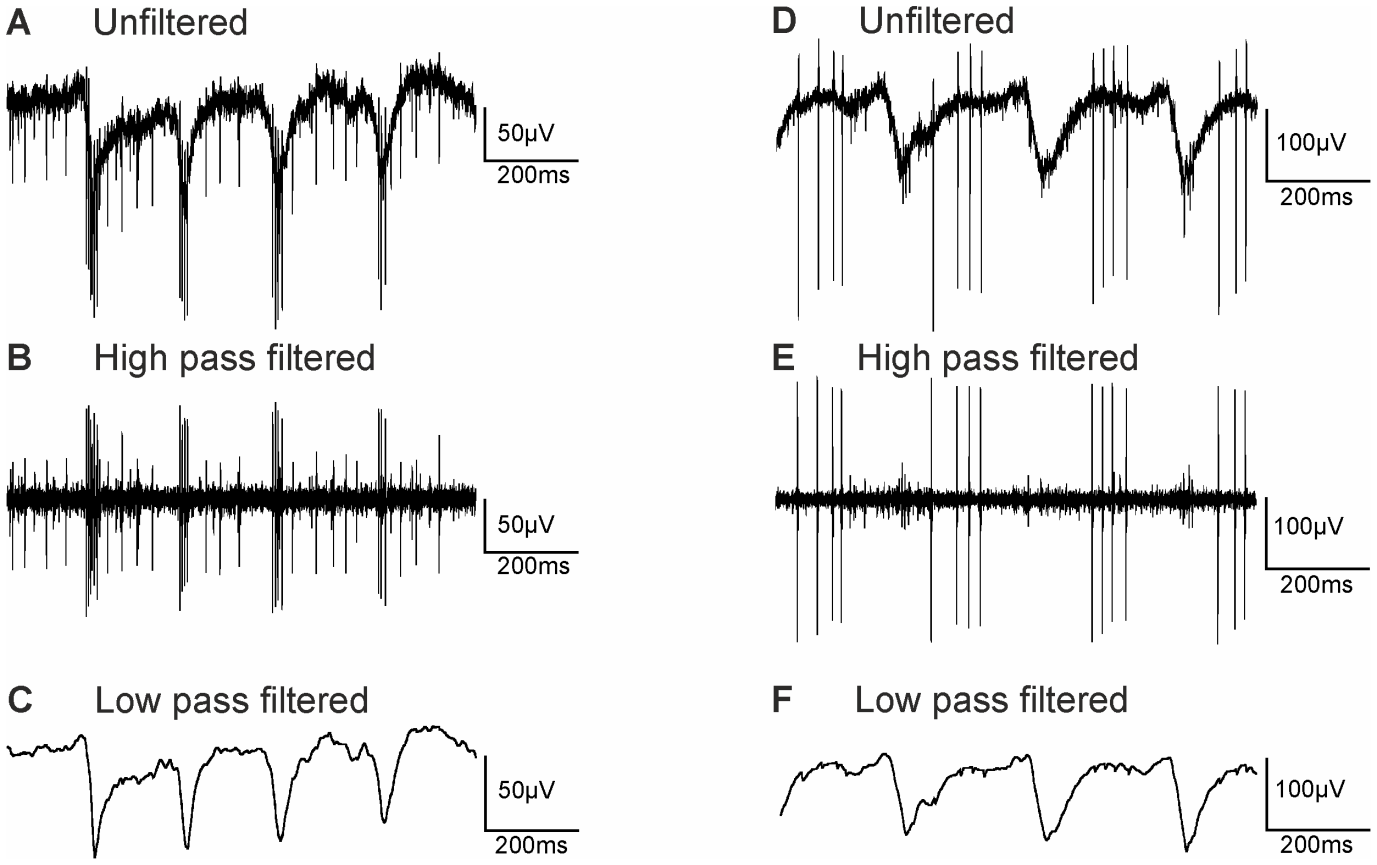
Rhythmic activity is observed in MEA recordings from *rd10* retina (A – C, and D – F). (A) Typical unfiltered recording of neural activity from a *rd10* retina (age 12 months). (B) Spontaneous spiking activity obtained after high-pass filtering (300 Hz cut off frequency). Large amplitude spikes occurred in rhythmic bursts with interburst intervals of ∼200 ms. Short amplitude spikes (from a different cell) were fired more regularly. (C) Local field potentials (LFPs) obtained after low pass filtering (50 Hz cut off frequency). Oscillations with a frequency of around 5 Hz were observed. (D) Unfiltered recording from another *rd10* retina (age 9 months). (E) High-pass filtered recording with spike firing during the positive-going or flat part of the LFP. (F) Low pass filtered recording.

We recorded spontaneous electrical activity from isolated *rd10* retinae of adult animals (P30– P360) using multi-electrode arrays. Fig. 2A shows a typical recording from one electrode displaying two different signal components (age of animal 12 months). First, fast voltage changes reflect extracellularly recorded action potentials of retinal ganglion cells that can be isolated using appropriate filter settings (Fig. 2B, high pass filter setting 300 Hz). In the lower frequency range (Fig. 2C, low pass filter setting 50 Hz) voltage modulations can be observed also known as slow wave components [15]. These local field potentials (LFPs) reflect changes in the extracellular ion composition that accompany neuronal activity. Oscillations in the LFPs were observed regularly in *rd10* retinae, but were never recorded from wt retinae under these experimental conditions (data not shown).

Ganglion cell spiking patterns could vary from cell to cell. In many instances, rhythmic ganglion cell spiking was observed. The recording in Fig. 2A - C displays spikes from at least two ganglion cells. The spikes with large amplitudes were fired in very short bursts that were phase locked to the minima of LFPs, while the spikes with smaller amplitude were more evenly distributed (Fig. 2B, filtered data). As displayed in Fig. 2D - F from another recording (age of animal 9 months), spikes could also appear phase locked to other phases of LFPs. In this example, spikes were not fired as short bursts but rather in groups of 3–4 spikes during the positive-going or flat component of the LFP.

Electrical activity recorded from *rd10* retinae differs strongly from that found in wt retinae, but is reminiscent of the electrical activity observed in *rd1* retinae. However, peaks in the LFPs occur at different frequencies. While in *rd1*, frequencies of 10–16 Hz were observed [14], [16], [23], we and others [16] mostly found basic frequencies of 3–4 Hz in *rd10*. In some cases, we also observed frequencies of 5–6 Hz in *rd10* retinae (see Fig. S1). In most instances, Fourier analysis revealed peaks with second or even higher harmonics (Fig. S1). For this study, LFPs were recorded from retinae of a total of 33 animals. Detailed results are provided in Table S1. In 9 animals aged 1 or 3 months, frequencies around 6 Hz were observed. In 24 animals aged from 4–12 months, frequencies around 4 Hz were observed. In our pharmacological experiments animal age was between 3 and 12 months. This means, that all rods and with few exceptions also cones had disappeared and remodelling as shown in Fig. 1 had taken place.

**Figure 3:**
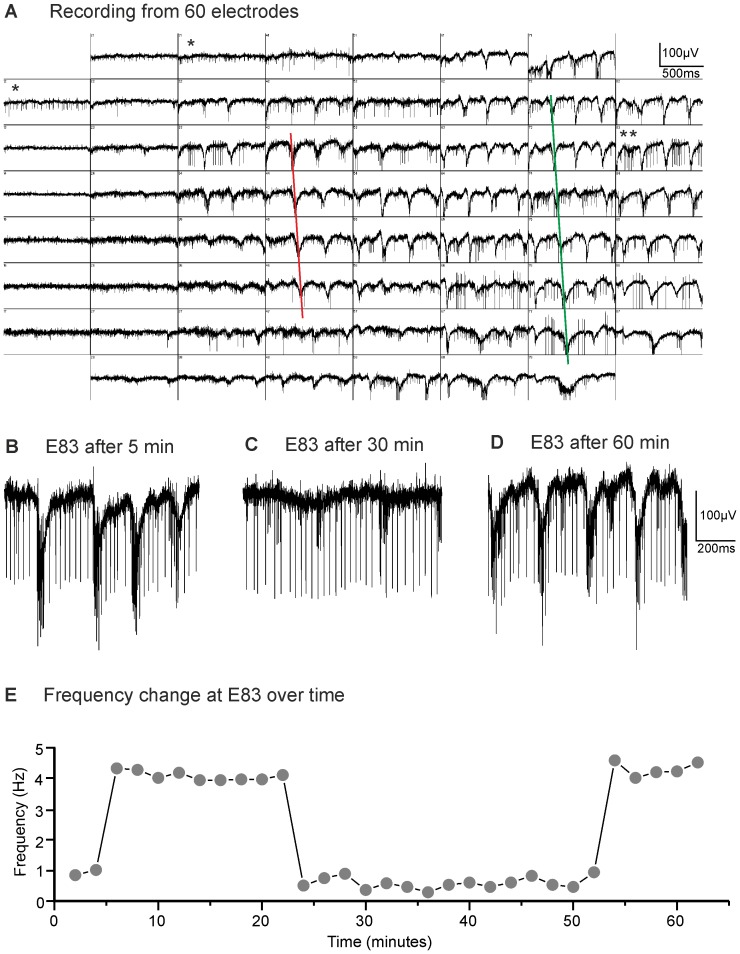
Spontaneous rhythmic electrical activity in *rd10* mouse retina can change. (A) Activity recorded simultaneously from 60 electrodes in an 8x8 matrix (age of animal: 9.5 months). Oscillations were observed in the majority of channels along with spiking activity. Some channels showed no oscillations (e.g. indicated by single asterisk). The phase of the oscillation seemed to be shifted from upper rows to lower rows of electrodes as indicated by red and green lines. (B–E) Recording of electrode 83 (E83, marked by two asterisks in A) obtained over time. Oscillations were visible during the first 22 min (B), vanished for the next 30 min, while spiking activity remained (C), and finally came back with similar frequency and shape (D) as before. (E) Frequency plotted vs time.

Spontaneous rhythmic activity has been reported for *rd1* and *rd10* by several studies. However, it is important to point out that oscillations are not always present. LFPs recorded in *rd10* retinae varied considerably from retina to retina, from electrode to electrode within one retina and even within one electrode over time. Fig. 3A shows a recording from 60 electrodes in one retina (age of animal 9.5 months). The distance between the electrodes was 100 µm. In several electrodes spikes were readily recorded, but no oscillations were observed (e.g. single asterisk). In most electrodes, however, strong oscillations could be recorded. Interestingly, the principal shape of the LFPs recorded at the different electrodes was very similar. Moreover, from upper to lower electrodes the phase of the LFPs seemed to be shifted. We marked the corresponding minima in the LFPs for electrodes 43 to 46 by a red line and for electrodes 72 to 77 by a green line. The two lines show the same slope. A simple way to interpret this phase shift is that the rhythmic activity is triggered at one site of the retina and then travels as a wave along the retina. By dividing the electrode distance by the time delay between the local minima at different electrodes we calculated the propagation velocity for the wave as 3.3 mm/s. This is slower than the velocity of wave propagation determined for *rd1* by Menzler and Zeck [17]. This could either reflect a difference between *rd1* and *rd10* retinae, or could depend on different recording conditions (experiments in our study were performed at room temperature, in the other study at 33–36°C).

LFPs recorded at one electrode could vary over time. Fig. 3B - D show recordings of electrode 83 (marked by two asterisks in Fig. 3A) taken 5, 30, or 60 min after the start of the experiment. In Fig. 3E the frequency of the oscillation obtained from the recording at this electrode was plotted over time for about one hour. Throughout the experiment, the retina was continuously superfused with oxygenized Ames solution without pharmacological agents at a constant rate. Oscillations were clearly visible for about 20 min (B, E), then vanished for about 30 min (C, E) and finally came back with a similar shape and frequency as observed during the first part of the experiment (D, E). These changes in the recording were not due to changes in the contact between electrodes and the retina, as ganglion cell spikes were recorded with the same amplitude at this electrode throughout the experiment. In summary, the results shown in Fig. 3 indicate that rhythmic electrical activity is not necessarily continuously generated but may depend on yet unknown physiological parameters.

### Rhythmic Electrical Activity Depends on Glutamatergic Input

The differences in the frequencies observed in LFPs from *rd1* and *rd10* retinae could indicate that the mechanisms underlying the rhythmic activity differ in the two animal models. While the origin of the rhythmic activity in *rd1* has not been identified unequivocally, certain evidence suggests that bipolar cells and amacrine cells may be involved. First, rhythmic changes in membrane potential were recorded from AII amacrine cells and from ON-cone bipolar cells in *rd1* mouse retina [14]. Second, oscillations as well as rhythmic spiking of ganglion cells were blocked by application of a mixture of the blockers for AMPA/kainate receptors and NMDA receptors CNQX and AP5 [12], [14], [15], [17], indicating that glutamatergic excitation most probably from cone bipolar cells is involved in the generation of rhythmic activity. Finally, blockers of the two inhibitory transmitters glycine and GABA used by the vast majority of amacrine cells seemed to modulate rather than abolish rhythmic electrical activity [15], [17]. In the following, we tested whether pharmacological modulation of rhythmic electrical activity in *rd10* mice would differ from that observed in *rd1*.

**Figure 4:**
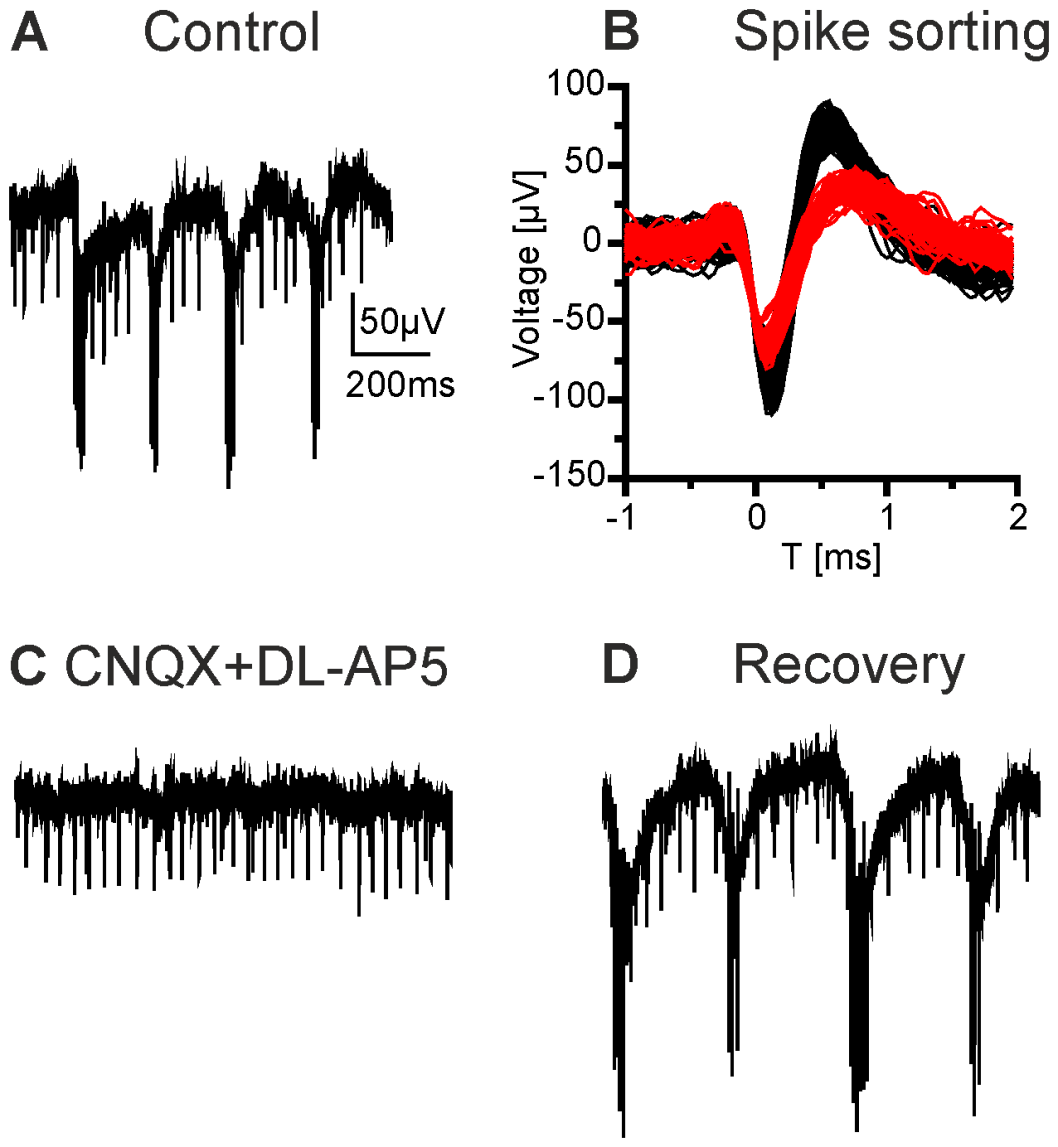
Glutamate receptor blockers CNQX and DL-AP5 abolish rhythmic electrical activity. (A) Unfiltered recording of an *rd10* retina displaying oscillations and spontaneous spiking activity (age 9 months). (B) Spike sorting of the recording shown in A. Spikes of two different amplitudes indicate activity from two ganglion cells. (C) Application of CNQX and DL-AP5 completely blocked the oscillations and bursts of large amplitude spikes. Small amplitude spikes remained unaffected. (D) The effect was reversible upon washout.

Superfusion of the retina with a mixture of CNQX and DL-AP5 abolished LFPs in *rd10* retinae in a reversible way (Fig. 4; age of animal 9 months). The recording in Fig. 4A shows rhythmic activity observed in the LFPs as well as spikes originating from two different cells. These spikes could be readily separated by conventional spike sorting algorithms (Fig. 4B). The spikes with the large amplitude were phase locked to the minima observed in LFPs, while the spikes with smaller amplitude were not. Upon application of CNQX/DL-AP5, oscillations in the LFPs were abolished (similar results were observed using CPP instead of DL-AP5, data not shown). Interestingly, the large amplitude spikes were also abolished while the small amplitude spikes remained. In a total of 9 experiments, we always observed that phase locked spikes vanished during application of CNQX/DL-AP5. In comparison to control recordings, remaining spike activity was on average reduced by 25%. Similar results were obtained in a total of 9 retinal pieces from 3 animals aged 7–9 months.

### Rhythmic Electrical Activity is Shaped by Inhibitory Input

**Figure 5:**
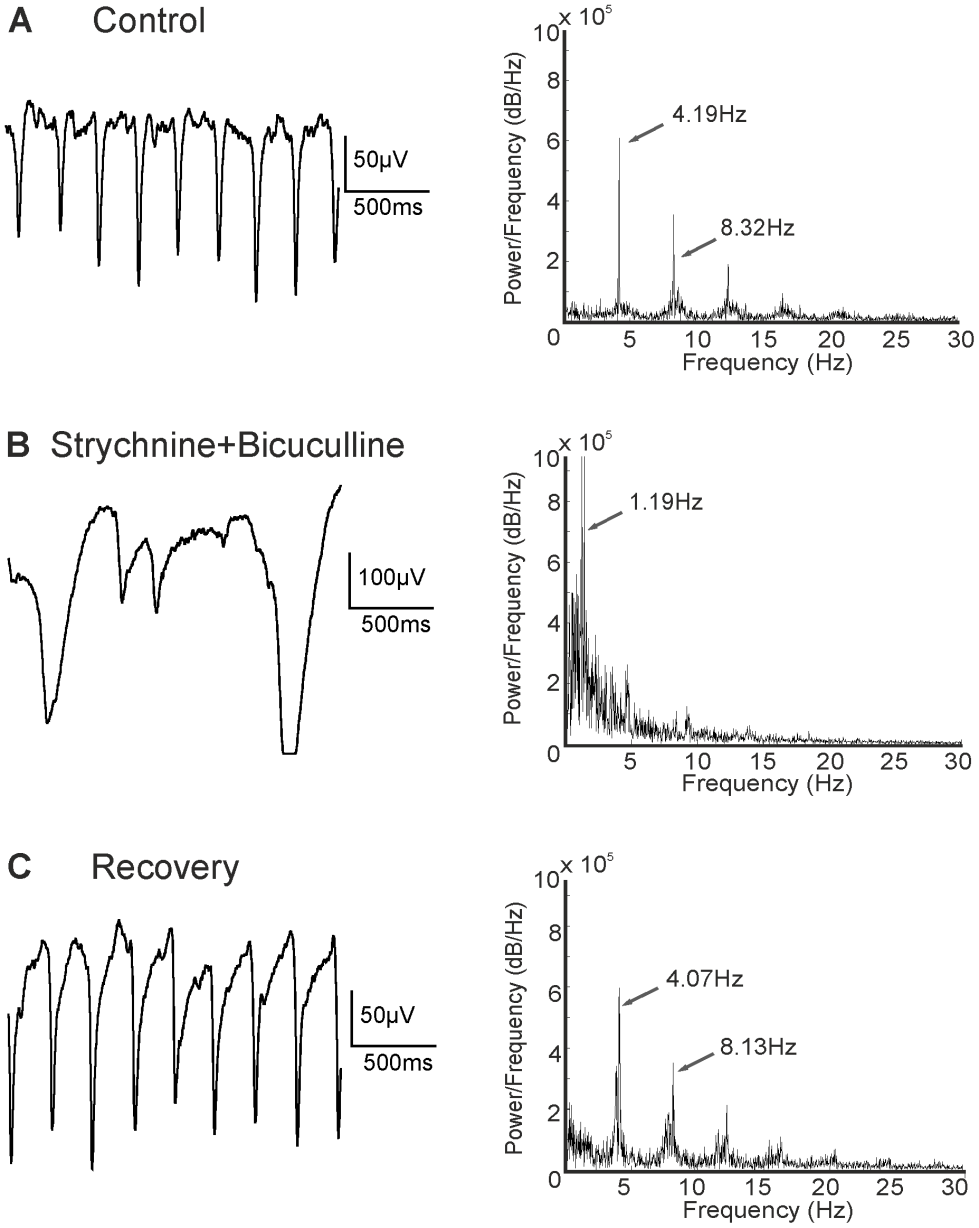
Effects of strychnine and bicuculline on rhythmic electrical activity. (A) Control recording (low pass filtered) displaying oscillations with a main frequency of ∼4 Hz (age 10 months). (B) Co-application of strychnine and bicuculline increased the LFP amplitude but reduced the frequency to ∼1 Hz. (C) The effect was fully reversible upon washout.

Application of the glycine receptor blocker strychnine or the GABA_A_ receptor blocker bicuculline alone hardly affected the spiking activity or the LFPs in *rd10* retinae (data not shown). However, co-application of both blockers strongly changed the LFP pattern. The amplitude of the oscillation was on average increased 2–4 fold (total of 15 retinal pieces from 10 animals aged from 6.5–10 months). Sometimes the oscillatory signal saturated the amplifier of our MEA system. The frequency under control conditions (4.14±0.58 Hz) was dramatically reduced to values of 0.95±0.3 Hz. The effect was reversible upon washout of the blockers (Fig. 5A – C; age of animal 10 months).

**Figure 6:**
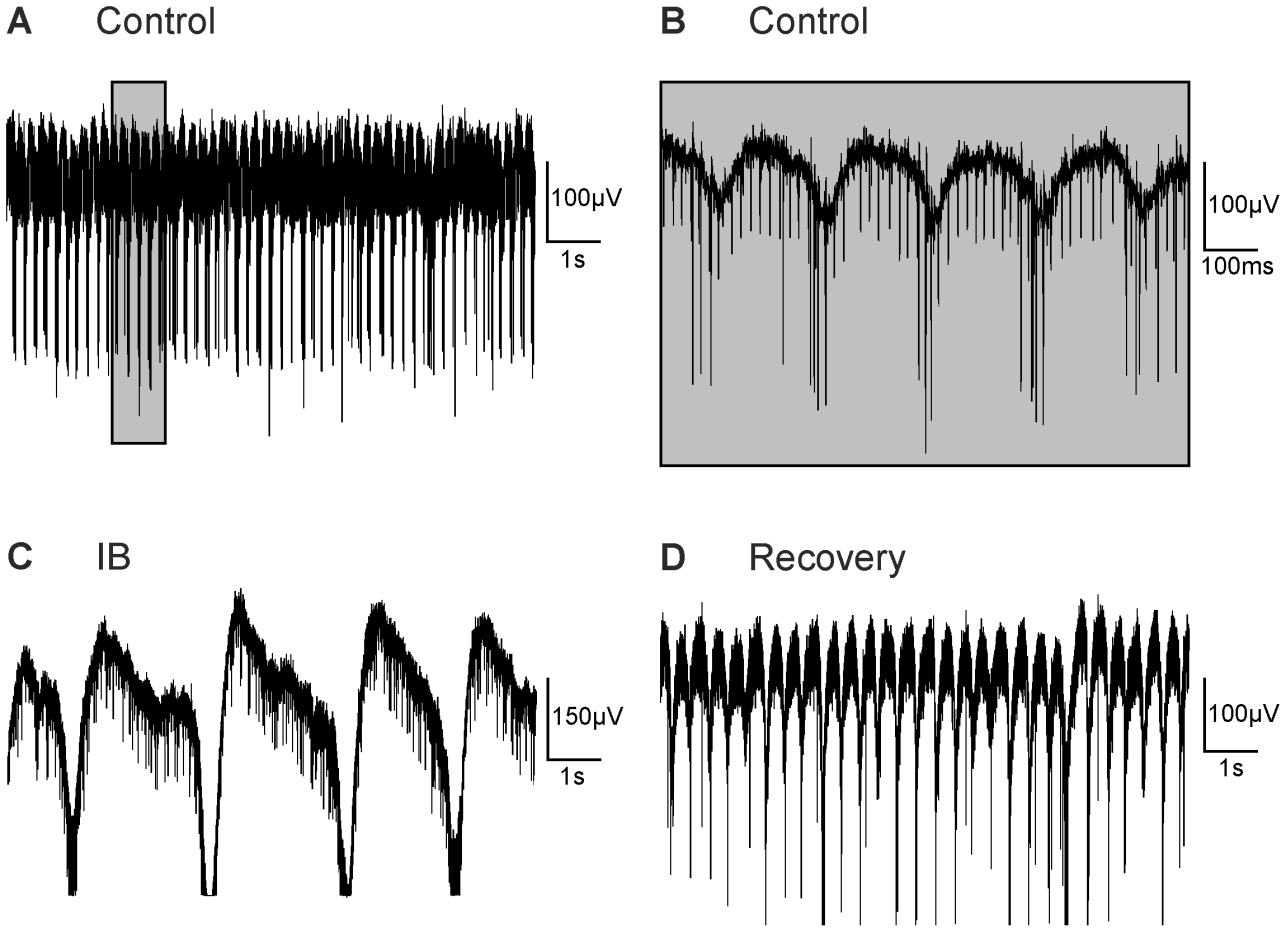
Effect of inhibitory blockers on the LFPs. (A) Unfiltered recording showing spontaneous spiking activity and oscillations (age 12 months). (B) A part of the recording from A (indicated by the grey box) at higher time resolution. (C) Co-application of strychnine, bicuculline, CGP 54626, and TPMPA (IB) reduced the frequency to around 0.4 Hz. The amplitude strongly increased, sometimes saturating the amplifier. (D) The effect was reversible upon washout.

**Figure 7:**
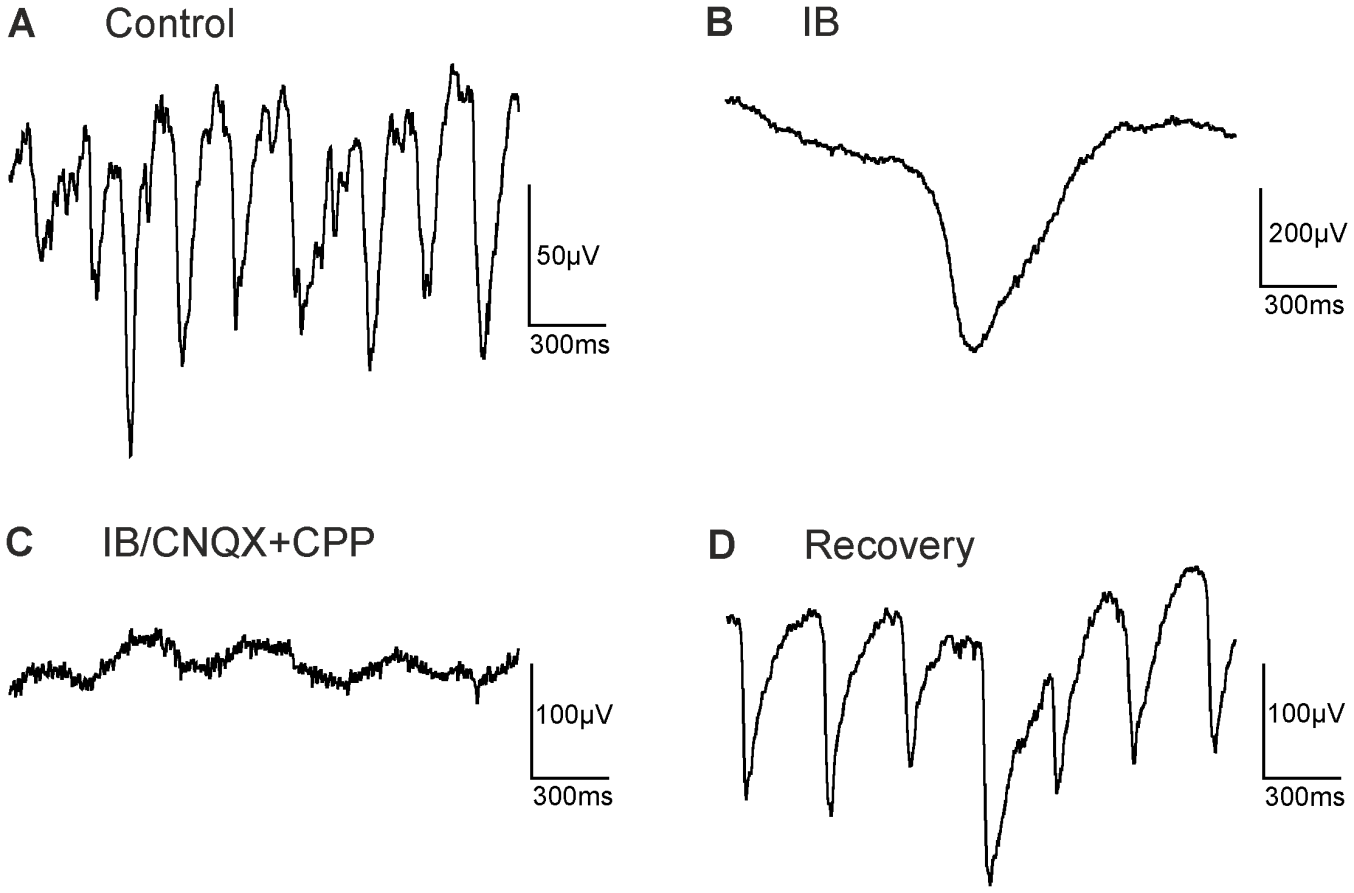
Glutamate receptor blockers abolish baseline fluctuations induced by inhibitory blockers. (A) Control recording (low pass filtered), showing LFPs with a frequency of ∼4 Hz (age 9 months). (B) Upon application of inhibitory blockers (IB = strychnine, bicuculline, TPMPA, CGP 54626), the LFP frequency was reduced to below 1 Hz. Note the change in the amplitude scale. (C) Application of glutamate receptor blockers (CNQX and CPP) nearly abolished baseline fluctuations. (D) Upon washout of all agents typical oscillations of ∼3 Hz were observed.

Apart from glycine receptors and GABA_A_ receptors, GABA_B_ and GABA_C_ receptors are also involved in synaptic inhibition in the retina [24]–[27]. Therefore, in a second set of experiments, we blocked all four inhibitory receptor types by a mixture of strychnine, bicuculline, CGP 54626, and TPMPA (Fig. 6; age of animal 12 months). Upon application of this cocktail, dramatic changes in the local field potentials occurred. The amplitude of the oscillation increased to values that partially saturated the amplifier (Fig. 6C). The frequency of the oscillation was reduced to values between 0.2 and 1 Hz. These effects were reversible upon washout (total of 9 retinal pieces from 8 animals aged 9–12 months). In wt retinae, this cocktail of blockers did not induce spontaneous rhythmic activity as observed in *rd10* retinae (data not shown). Blockade of glutamate receptors abolished the effect of the inhibitory blockers (Fig. 7; age of animal 9 months). The large fluctuations in the baseline observed during the inhibitory cocktail (Fig. 7B) were nearly absent during application of glutamate receptor blockers (Fig. 7C; 4 retinal pieces from 4 animals aged 9–12 months).

L-APB is an agonist at mGluR6, the metabotropic glutamate receptor present on both rod and cone ON-bipolar cells. L-APB hyperpolarizes ON-bipolar cells by starting a G-Protein dependent pathway that leads to the closure of cation channels, most likely TRPM1 [28]–[35]. If cone ON-bipolar cells are involved in the generation of rhythmic electrical activity, one might postulate that L-APB affects LFPs. Application of L-APB did not yield unequivocal results. In some cases, the LFP amplitude was slightly diminished. In most cases, however, L-APB affected neither amplitude nor frequency of the oscillations in a clear cut way (data not shown). This is probably due to lower expression of mGluR6 associated with the fact that during retinal remodelling mGluR6 becomes distributed over the somata and axons of bipolar cells (Fig. 1H) and may not effectively couple to its downstream signalling cascade [36].

### Rhythmic Electrical Activity in rd10 Depends on Gap Junctions and is Modulated by HCN Channels

**Figure 8:**
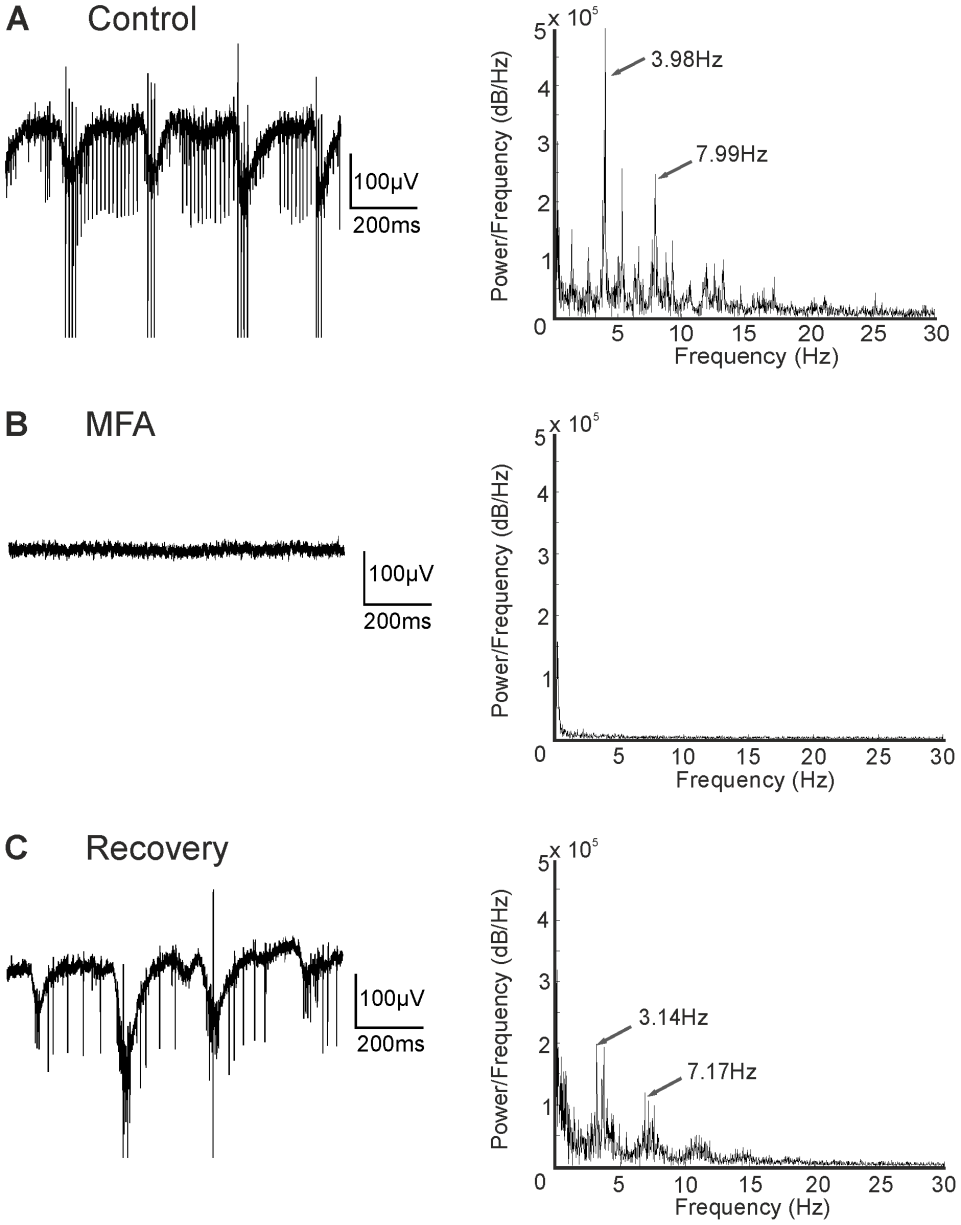
The gap junction blocker MFA abolishes rhythmic electrical activity. (A) Unfiltered baseline, showing spontaneous activity and LFPs with a frequency of ∼4 Hz (age 9.5 months). (B) Application of MFA blocked all spontaneous activity. (C) The effect was reversible upon washout.

**Figure 9:**
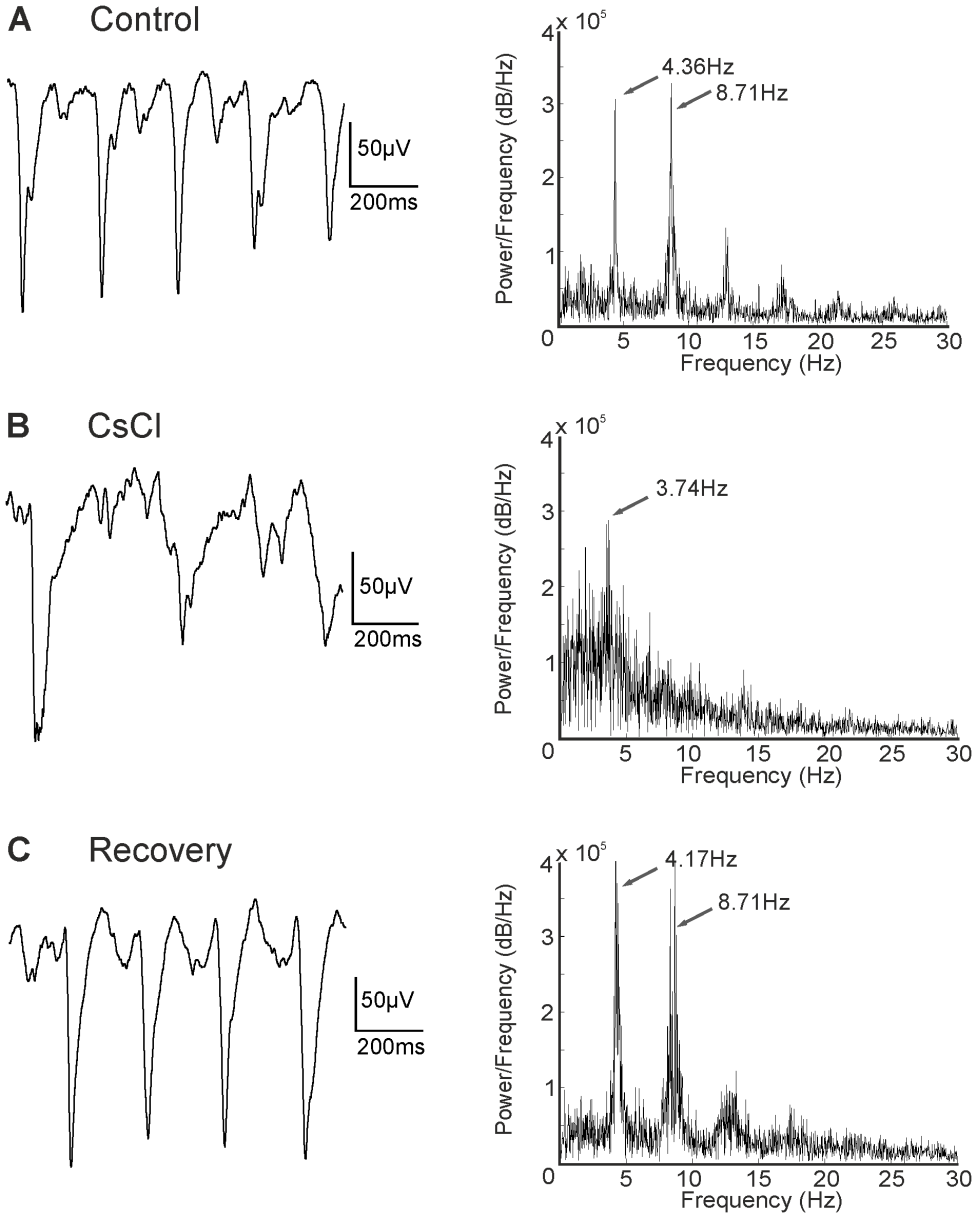
HCN channel blocker reduces rhythmic electrical activity. (A) Low pass filtered recording, frequency ∼4 Hz (age 9.5 months). (B) Blocking HCN channels with CsCl abolished the second harmonic peak and slightly reduced the frequency of the first peak. (C) Recovery.

AII amacrine cells are electrically coupled to each other and to cone ON-bipolar cells [37]. Application of the gap junction blocker MFA totally abolished spontaneous rhythmic activity in *rd1* retina [12], [14], [17], [38]. We observed that this was also true for *rd10* retina (Fig. 8; age of animal 9.5 months; similar results were found in a total of 9 retinal pieces from 4 animals aged 9–10 months). Moreover, ganglion cell spiking was also totally abolished. Both effects were reversible upon washout (Fig. 8A – C). In the retina, all four isoforms of the hyperpolarization-activated and cyclic nucleotide-gated (HCN) channels that can function as pacemaker channels are expressed [39]–[42]. HCN channels, in particular when expressed in bipolar cells, might contribute to the rhythmic electrical activity observed in *rd1* retina [12] and *rd10* retina. We blocked HCN channels in two ways: by application of 3 mM Cs^+^ (6 retinal pieces from 3 animals aged 9.5–10 months) and by application of ZD7288 (4 retinal pieces from 1 animal aged 9 months; data not shown). Both blockers affected spontaneous rhythmic activity in the same way: rhythmic activity persisted but the second harmonic frequency component disappeared while the basic frequency was reduced (Fig. 9A – C; age of animal 9.5 months). On average frequencies changed from 4.65±0.51 Hz under control conditions to 2.78±0.69 Hz during HCN channel blockage.

## Discussion

In the recent years, many studies have been performed on the retina of the *rd1* mouse, describing in detail the photoreceptor degeneration, the rewiring that takes place in the inner retina as well as changes in functional properties of retinal neurons [8], [9]. However, as discussed earlier, due to the delayed and slower degeneration process, *rd10* may mimic human RP better than *rd1* and, therefore, may be a better model [10], [11]. In both *rd1* and *rd10* retinae spontaneous rhythmic activity was observed. Rhythms display frequencies of 10–16 Hz in *rd1*, but of 3–7 Hz in *rd10*. Most attempts to decipher the origin of spontaneous rhythmic activity were performed in *rd1* [12], [14], [15], [17], [43]. However, as *rd10* is increasingly being used to study the process of retinal degeneration as well as a model to develop experimental therapies like stimulation of retinal ganglion cells by implants or by light-driven channels, it is important to understand the mechanisms underlying the spontaneous rhythmic activity. We, therefore, investigated whether the mechanisms suggested to elicit rhythmic activity in *rd1* also apply to *rd10*. We found, that despite the differences observed in the frequencies, the origin of rhythmic electrical activity in *rd1* and *rd10* seems to be quite similar.

In principle there are several possibilities how rhythmic activity could be generated. First, excitatory cells like bipolar cells might display endogenous rhythmic activity. Second, a continuous excitatory drive from bipolar cells could be modulated by inhibition that arises from rhythmic activity of amacrine cells. Indeed, spontaneous activity in several types of amacrine cells has been reported [44], [45]. Finally, both mechanisms might contribute to rhythmic activity. We (Fig. 4) and others found that in both, *rd1* and *rd10,* blockage of ionotropic glutamate receptors abolishes oscillations, indicating that glutamatergic neurons – most likely bipolar cells - are a major drive for rhythmic activity [14], [15], [17]. In *rd10* we found that during blockade of glycinergic receptors and GABA_A_ receptors oscillations persisted. However, the frequency was reduced to around 1 Hz while the amplitude of the oscillations was increased 2–4 fold. Additional blockage of GABA_B_ and GABA_C_ receptors further reduced the frequency to 0.2–1 Hz and increased the LFP amplitude. These results indicate that rhythmic activity in *rd10* arises even in the absence of inhibitory input and, therefore, most likely originates from bipolar cell activity. Yet, inhibitory mechanisms modulate both frequency and amplitude of the oscillations. Blockade of ionotropic glutamate receptors abolishes the effects of inhibitory receptor blockers (Fig. 7). This might indicate that inhibitory receptor blockade acts presynaptically to the glutamate receptors, and hence on the bipolar cells. On the other hand, activity of amacrine cells seems to depend strongly on glutamatergic input [12], [14]. In this case, inhibitory blockers would be without effect if glutamatergic input to amacrine cells is blocked.

For *rd1*, results with inhibitory blockers are less consistent. Menzler and Zeck [17] described an overall reduction in LFP frequency and an increase in LFP amplitude during application of bicuculline/strychnine, yet do not present numbers. Strychnine alone had a stronger effect than bicuculline. However, judging from their Supplementary Fig. 2, strychnine barely changed the LFP frequency. Ye and Goo [15] found that strychnine and picrotoxin, another blocker of GABA_A_ receptors, either alone or in combination increased the LFP amplitude 2–3 fold while the frequency was reduced to values around 4–5 Hz (as judged from their Fig. 4). This corresponds to a roughly twofold decrease in frequency in *rd1*, while in *rd10* frequency was reduced 3–5 fold in the presence of strychnine and bicuculline and even stronger if all inhibitory receptors were blocked (Fig. 6).

In recordings obtained from bipolar cells and AII amacrine cells of *rd1* retina, spontaneous oscillations in membrane potential were observed that strongly resembled rhythmic activity recorded using MEAs [12], [14]. The authors suggest that rhythmic activity originates from the tight interaction between ON-cone bipolar cells and AII amacrine cells. AII amacrine cells contact ON-cone bipolar cells via electrical synapses in form of gap junctions [46]–[50]. In fact, MFA that was shown to block gap junctions in the retina [51], [52] abolished oscillations in both *rd1* [12], [14], [17] and *rd10* (present study; [38]). Interestingly, ON-bipolar cells express hyperpolarization-activated and cyclic nucleotide-gated (HCN) channels [39]–[42]. In several systems, HCN channels function as pacemaker channels [53], [54] and could, therefore, be the source of spontaneous rhythmic activity in ON-bipolar cells. However, blocking HCN channels using either Cs^+^ or ZD7288 did not abolish oscillations in the membrane potential of ON-cone bipolar cells in *rd1* retinae [12]. HCN channel blockers reduced the frequency from 10 Hz to around 6 Hz, but increased the power at the peak oscillatory frequency by more than 700%. We did not observe such an increase in power in *rd10* retinae. In *rd10*, Cs^+^ and ZD7288 mostly reduced the second peak of the power spectrum but only slightly changed the frequency of the first peak (Fig. 9).

In summary, despite the differences in the process of degeneration and the different frequencies of spontaneous rhythmic activity in *rd1* and *rd10*, we only found small differences in the pharmacological modulation of rhythmic activity in both models. This indicates that the cellular origin of the spontaneous activity may be quite similar in *rd1* and *rd10.* Could the difference in frequencies between *rd1* and *rd10* be attributed to different genetic backgrounds? We found that in *rd10* the frequency observed in LFP oscillation is modulated by a variety of ion channels, amongst them HCN channels, glycine receptors, and GABA receptors. Differences in the expression level of any of these channels between mice of different backgrounds might, therefore, affect the frequency of the oscillations. Our animals carried the *rd10* mutation in a C57BL/6 background. Trenholm and colleagues recorded oscillations in the membrane potential of AII amacrine cells and ON-cone bipolar cells in retinal slices from animals carrying the *rd1* mutation in the same background. They found frequencies of around 15 Hz (see e.g. their Fig. 1) [12]. These values are very different from those obtained in the present study for *rd10*, but consistent with data obtained by the same authors from animals carrying the *rd1* mutation in a different genetic background and with previous reports on *rd1* retinae using MEA recordings [13]–[16], [55].

We (Fig. 3) and others provided evidence that rhythmic electrical activity might spread across the retina in form of a wave [17]. This is reminiscent of waves of retinal activity observed during the early postnatal period before eye opening (e.g. [56], [57]) and of the propagation of electrical excitation in the heart. It is not entirely clear why spontaneous activity originating at one spot in the retina should dominate oscillations over a larger retinal area, but once generated the pacemaker wave could easily spread across the retina through the gap junctional network between the AII amacrine cells [37]. Further studies will have to identify the mechanism underlying the generation of such waves and will have to address the question why robust spontaneous activity disappears entirely for longer time and comes back (Fig. 3).

Why does spontaneous rhythmical activity originate at all in the retina of *rd1* and *rd10* mice? Spontaneous rhythmic activity could result from substantial remodelling and rewiring processes described in retinal tissue upon photoreceptor degeneration. Such processes could transform the retina into a self-signalling neuronal network [6]–[10], [58]. Moreover, several lines of evidence suggest that changes of the functional properties of bipolar cells occur at early stages of degeneration when photoreceptors are still present and long before substantial remodelling has taken place. These changes include the aberrant expression of ionotropic glutamate receptors and the loss of expression or reduced activation of metabotropic glutamate receptors on ON-bipolar cells as well as the increase of GABA mediated currents [3], [6], [24], [36], [59], [60]. Such changes could explain why oscillations can be observed in *rd10* mice at postnatal week 2, when photoreceptors are still present and no remodelling has occurred [61]. On the other hand, there is evidence that rhythmic activity in the retina does not depend on complex rewiring processes or degeneration-induced changes of bipolar cell physiology. Trenholm et al. [12] reported that in wild type retina, blockage of photoreceptor input by application of NBQX and APB induces membrane oscillations in both ON-cone bipolar cells and AII amacrine cells similar to those observed in *rd1* retina. This would suggest that the lack of photoreceptor input is sufficient to trigger oscillations in the retina. Clearly, while over the last years we have gained considerable insight into the process of retinal degeneration, several important issues still need clarification in further studies.

## Materials and Methods

### Animals

Wildtype animals of the strain C57BL/6 were obtained from Charles River. *Rd10* mice were bred locally from breeding pairs obtained from Jackson (strain name: B6.CXB1-*Pde6b* ^rd10^/J). In this line the *rd10* mutation was backcrossed onto the C57BL/6J background for 5 generations before intercrossing to homozygosity. All animals were kept on a 12 h light/dark cycle with food and water ad libitum. All experiments were performed in accordance with the German Law for the Protection of Animals and after approval was obtained by the regulatory authorities, the Forschungszentrum Jülich and the Landesamt für Natur, Umwelt und Verbraucherschutz of the land North-Rhine Westfalia.

### Immunohistochemistry

For immunohistochemistry, animals were deeply anesthetized with isoflurane and killed by decapitation. The eyes were enucleated and opened by an encircling cut at the limbus. The retinae in the eyecup were immersion-fixed for 30 min in 4% paraformaldehyde (PA) in 0.1 M phosphate buffer (PB; pH 7.4) at room temperature and washed in PB several times. Tissue was incubated in 10% sucrose in PB for 1 h, followed by 30% sucrose in PB overnight. The retina was flat embedded and frozen in optimal cutting temperature (OCT) compound (NEG-50, Richard Allen Scientific, Thermo Fisher Scientific, Germany). Vertical sections (i.e. perpendicular to the retinal layers, 20 µm thick) were cut on a cryostat (HM 560 CryoStar; MICROM; Walldorf; Germany) and collected on Superfrost Plus slides (Menzel, Braunschweig, Germany). Sections were pre-treated with blocking solution (5% Chemiblocker (Chemicon, Hofheim, Germany), 0.5% Triton-X100, 0.05% NaN_3_ in PB) for 1 hour, followed by incubation with primary antibodies over night, diluted in the same solution. Sections were washed in PB and incubated in secondary antibodies diluted in 5% Chemiblocker, 0.5% Triton-X100 in PB for 1 h, washed in PB and coverslipped with Aqua Polymount (Polysciences, Eppelheim, Germany). Sections were examined with a confocal laser scanning microscope (Leica TCS SP5, Leica Microsystems, Heidelberg, Germany) with 63x/1.4 oil immersion lenses. Images were processed and printed with Adobe Photoshop. Primary antibodies included AB5585 (anti-recoverin polyclonal antibody, raised in rabbit, 1∶2000, Chemicon, Germany); AB5405 (anti-opsin red/green, raised in rabbit, 1∶800, Chemicon, Germany); PKCα (anti-protein kinase Cα, raised in rabbit, 1∶4000; Santa Cruz Biotechnology, Inc.); mGluR6 (anti-GRM6, raised in rabbit, 1∶1000, Sigma, Germany); CabP (anti-calbindin 28k, raised in mouse, 1∶1000, Sigma, Germany). Secondary antibodies included donkey anti-rabbit Cy2 (1∶400, Dianova, Germany), donkey anti-rabbit Cy3 (1∶500, Dianova, Germany), goat anti-rabbit A488 (1∶500, Invitrogen, Germany), and donkey anti-mouse Cy3 (1∶100, Dianova, Germany).

### Multi-electrode Arrays (MEA) and Data Recording

3D MEAs containing 60 platinum electrodes (conical shape, diameter: 30 µm or 40 µm) on a glass substrate (Qwane Biosciences, Lausanne, Switzerland) with 8x8 matrix without corner electrodes, were used for recording of local field potentials (LFP) and spiking activities from ganglion cells. Spacing between electrodes was either 100 µm or 200 µm. Impedances of the electrodes were 600–900 kΩ (Ø 30 µm) and 250–450 kΩ (Ø 40 µm). The MEA60 data acquisition system (MC_Card, Multichannel system, Reutlingen, Germany) consisted of a RS-232 interface, an integrated preamplifier and MEA 1060 bandpass filter (amplification gain: 1200), and a personal computer. The waveforms were recorded with the sampling frequency rate of 25 kHz/channel. The data were later converted to ASCII files by MC_Data for further analysis with OriginPro8 and by custom made MATLAB scripts.

### Tissue Preparation

Retinae of wild type (adult) and *rd 10* (postnatal day 30–12 months) mice were prepared for MEA recordings. Briefly, the mouse was deeply anesthetized with isoflurane and killed by decapitation. The eyeballs were enucleated and retinae were isolated. The retinae were cut into two halves and one half was mounted with ganglion cells towards the electrode side of the MEA. MEAs were pre-treated in a plasma cleaner (Diener Electronic GmbH+Co. KG, Germany) and coated with Poly-D-lysine hydrobromide (PDL, Sigma, Germany). The retinal preparation was maintained in carbonate-buffered AMES solution, bubbled with 95% O_2_+5% CO_2_ at a pH of ∼7.4. All pharmacological agents were dissolved in oxygenated AMES buffer and delivered to the retina by continuous perfusion at a flow rate of 3 ml/min. All results shown are from experiments performed at room temperature (RT). Oscillations at RT (4–6 Hz) were similar to those found in own preliminary experiments carried out at 32°C (5–7 Hz) and to those reported for *rd10* at 32°C (4–7 Hz) [16]. However, in our hands, recordings at RT showed more stable oscillations and were, therefore, chosen for the long lasting recordings performed in this study.

### Pharmacology

The following pharmacological agents were used to study the origin of intrinsic neuronal oscillations: L-(+)-2-Amino-4-phosphonobutyric acid (L-APB), 100 µM, agonist at mGLUR6; 6-cyano-2,3-dihydroxy-7-nitro-quinoxaline-2,3-dione disodium (CNQX), 20 µM, AMPA/kainate receptor antagonist; DL-2-Amino-5-phosphonopentanoic acid (DL-AP5), 50 µM, NMDA receptor antagonist (D-AP5 is a strong NMDA receptor antagonist, while L-AP5 has been reported to be a weak agonist at glutamate receptors; we found no difference in the effect of the mixtures CNQX/DL-AP5 and CNQX/R-CPP, respectively); 3-((*R*)-2-Carboxypiperazin-4-yl)-propyl-1-phosphonic acid (R-CPP), 40 µM, NMDA receptor antagonist; strychnine, 10 µM, glycine receptor antagonist; bicuculline, 30 µM, GABA_A_ receptor antagonist; [*S*-(R*,R*)]-[3-[[1-(3,4-Dichlorophenyl)ethyl]amino]-2-hydroxypropyl](cyclohexylmethyl) phosphinic acid (CGP 54626), 2 µM, GABA_B_ receptor antagonist; 1,2,5,6-Tetrahydropyridin-4-yl)methylphosphinic acid (TPMPA), 100 µM, GABA_C_ receptor antagonist; meclofenamic acid (MFA), 100 µM, gap junction blocker; cesium chloride (CsCl), 3 mM, HCN channel blocker, and 4-Ethylphenylamino-1,2-dimethyl-6-methylaminopyrimidinium chloride (ZD7288), 100 µM, HCN channel blocker.

## Supporting Information

Figure S1 **Different frequencies can be observed in LFP oscillations in** ***rd10*** **.** Unfiltered recordings of different *rd 10* retinae (left) with Fast Fourier Transformations (FFT; right). (A) Main frequency at ∼3 Hz with a second harmonic frequency at ∼6 Hz (age 12 months). (B) Main frequency at ∼5 Hz and second peak at ∼10 Hz (age 8 months).
(TIF)

Table S1 **Frequencies observed in LFP oscillations in** ***rd10*** **.** Experiments were performed on a total of 33 animals ranging from 1–12 months. The group aged 9 months also includes the animals aged 9.5 months. The frequency of oscillations was slightly higher in animals between 1 and 3 months. In animals aged 4–12 months, little variation in the frequency was observed. Frequency is given as mean ± standard deviation (SD).
(DOCX)
