# Supplementary material for: Pharmacological Analysis of Intrinsic Neuronal Oscillations in rd10 Retina
Source: PLoS One. 2014 Jun 11;9(6):e99075. doi: 10.1371/journal.pone.0099075 (PMC4053359; doi:10.1371/journal.pone.0099075)
Supplement: Table S1 — Frequencies observed in LFP oscillations in rd10 . Experiments were performed on a total of 33 animals ranging from 1–12 months. The group aged 9 months also includes the animals aged 9.5 months. The frequency of oscillations was slightly higher in animals between 1 and 3 months. In animals aged 4–12 months, little variation in the frequency was observed. Frequency is given as mean ± standard deviation (SD). (DOCX) [file pone.0099075.s002.docx]

|  | \| **Age of animals (Months)** \| **Frequency (Hz)**  **mean ± SD** \| **Number of retinal pieces** \| **Number of animals and (number of pieces per animal)** \| \| --- \| --- \| --- \| --- \| \| 1 \| 6.21 ± 0.94 \| 12 \| 6 (2 each) \| \| 3 \| 6.08 ± 0.53 \| 7 \| 3 (2,2,3) \| \| 4 \| 4.14 ± 0.35 \| 4 \| 2 (2 each) \| \| 6 \| 4.01 ± 0.08 \| 3 \| 2 (1,2) \| \| 7 \| 4.21 ± 0.54 \| 7 \| 2 (3,4) \| \| 8 \| 4.23 ± 0.65 \| 9 \| 5 (2,2,2,2,1) \| \| 9 \| 4.40 ± 0.40 \| 15 \| 5 (3 each) \| \| 10 \| 4.31 ± 0.73 \| 5 \| 3 (2,2,1) \| \| 11 \| 4.03 ± 0.20 \| 2 \| 2 (1) \| \| 12 \| 3.58 ± 0.64 \| 3 \| 3 (1) \| |  |  |
| --- | --- | --- | --- | --- | --- | --- | --- | --- | --- | --- | --- | --- | --- | --- | --- | --- | --- | --- | --- | --- | --- | --- | --- | --- | --- | --- | --- | --- | --- | --- | --- | --- | --- | --- | --- | --- | --- | --- | --- | --- | --- | --- | --- | --- | --- | --- | --- |
